# Do the teaching, practice and assessment of clinical communication skills align?

**DOI:** 10.1186/s12909-024-05596-8

**Published:** 2024-06-01

**Authors:** Sari Puspa Dewi, Amanda Wilson, Robbert Duvivier, Brian Kelly, Conor Gilligan

**Affiliations:** 1 Department of Public Health, Faculty of Medicine, Universitas Padjadjaran, Jatinangor Km. 21 Sumedang West Java, Bandung, Indonesia; 2 School of Medicine and Public Health, The University of Newcastle, Newcastle, New South Wales Australia; 3 School of Medicine and Psychology, Australian National University, Canberra, Australia; 4 School of Nursing and Midwifery, The University of Technology Sydney, Ultimo, Australia; 5 Parnassia Psychiatric Institute, The Hague, The Netherlands; 6 Center for Education Development and Research in Health Professions (CEDAR), Faculty of Medical Sciences, University Medical Center Groningen, University of Groningen, Groningen, The Netherlands; 7 Faculty of Health Sciences & Medicine, Bond University, Gold Coast, Australia

**Keywords:** Communication skills, Constructive alignment, Learning environment, SDG 4: quality education

## Abstract

**Background:**

Evidence indicates that communication skills teaching learnt in the classroom are not often readily transferable to the assessment methods that are applied nor to the clinical environment. An observational study was conducted to objectively evaluate students’ communication skills in different learning environments. The study sought to investigate the extent to which the communication skills demonstrated by students in classroom, clinical, and assessment settings align.

**Method:**

A mixed methods study was conducted to observe and evaluate students during the fourth year of a five-year medical program. Participants were videorecorded during structured classroom ‘interactional skills’ sessions, as well as clinical encounters with real patients and an OSCE station calling upon communication skills. The Calgary Cambridge Observational Guides was used to evaluate students at different settings.

**Result:**

This study observed 28 students and findings revealed that while in the classroom students were able to practise a broad range of communication skills, in contrast in the clinical environment, information-gathering and relationship-building with patients became the focus of their encounters with patients. In the OSCEs, limited time and high-pressure scenarios caused the students to rush to complete the task which focussed solely on information-gathering and/or explanation, diminishing opportunity for rapport-building with the patient.

**Conclusion:**

These findings indicate a poor alignment that can develop between the skills practiced across learning environments. Further research is needed to investigate the development and application of students’ skills over the long term to understand supports for and barriers to effective teaching and learning of communication skills in different learning environments.

**Supplementary Information:**

The online version contains supplementary material available at 10.1186/s12909-024-05596-8.

## Background

Doctors’ communication skills are among the most essential elements of effective patient care [1, 2]. Studies show a clear link between the quality of explanations provided to patients and their health outcomes, including reduced pain, enhanced quality of life, improved emotional health, symptom alleviation, and better adherence to treatment plans [1, 3, 4]. Recognizing its significance, medical councils and accreditation bodies worldwide prioritize effective communication as a core competency for healthcare practitioners [5–7]. Experts agree that communication skills can be effectively taught and learnt [8–13]. Patients treated by doctors who have undergone communication skills training exhibit 1.62 times higher treatment adherence [4]. Furthermore, doctors trained in communication have better patient interactive processes and outcomes (information gathered, signs and symptoms relieved, and patient satisfaction) compared to those without such training [14].

Diverse medical consultation models have emerged, drawing from a spectrum of frameworks that prioritize tasks, processes, outcomes, clinical competencies, doctor-patient relationship, patients’ perceptive of illness, or a combination of these elements [15]. These models serve as frameworks conducive to structuring the teaching and learning of communication skills, delineating both content and pedagogical approaches. Numerous research endeavours have assessed these models’ applicability in clinical and educational settings [16, 17]. Medical schools can leverage these models to articulate learning objectives and tailor teaching strategies within their curricula accordingly. Furthermore, the model can serve as framework for evaluating communication skills and the effectiveness of training interventions. The adoption of such models across these diverse educational contexts however, appears to be inconsistent.

Communication skills teaching and learning typically commences in classroom or simulation settings before students transition to clinical practice. Classroom sessions involve simulation-based role-play exercises with peers and simulated patients [18, 19], while clinical environments offer opportunities for real patient interactions in various healthcare settings [20]. This structured learning process aims to develop students’ ability to conduct effective, patient-centered medical consultations across diverse clinical scenarios and handle challenging situations [20, 21]. Students’ ability to communicate with patients is commonly assessed using Objective Structural Clinical Examination (OSCE) [22], in which students are observed during an interaction with simulated patient in a set time and evaluated using a standardised rating form.

Constructive alignment, a crucial concept in education, refers to “aligning teaching methods, as well as assessment, to the intended learning outcomes” [23]. In medical education, this alignment ensures that students achieve desired learning outcomes related to communication skills necessary for delivering patient-centered care in diverse contexts [24]. Communication skills include the process of exchanging messages and demonstrating empathic behaviour while interacting with patients and colleagues to deliver patient-centred care in a range of contexts [25]. Learning clinical communication skills is complex and nuanced but should be subjected to the scrutiny and planning associated with constructive alignment along with other curricula elements. Both approaches to learning, and application of clinical communication skills often requires students to be creative and flexible in applying their skills to different contexts and patient conditions [19], and to be committed to ongoing development and improvement of their communication in practice [26, 27].

The achievement of such constructive alignment, however, remains an elusive goal in many medical schools, with challenges in aligning the communication skills learnt, modelled, and applied in different learning environments and assessment contexts [28–32]. Evidence indicates that the skills, suggested structures, and processes learnt in the classroom are not always transferred in the clinical environment [20, 33, 34]. Differences in learning processes exist between settings, and particularly in the transition from classroom to clinical environment, associated with heavy workloads, different teaching and assessment methods, students’ uncertainty about their role, and adaptation to a more self-directed learning style [35].

Although structured approaches to medical consultations, such as the Calgary-Cambridge Observational Guides (CCOG) [19] are often taught by classroom educators, teaching, feedback and modelling in clinical environment often does not align with this [20, 36]. Further, these structures are not always reflected in the rating schemes used to assess OSCE performance [37], or the structure of OSCE stations in which communication is not the only skill being assessed. OSCE stations differ in purpose and focus from those designed to assess communication as it is integrated within broader clinical tasks, to those which focus more specifically on communication [38], but time limited OSCE stations rarely reflect the true entirety of a complex clinical task [39]. Recent reviews indicate that OSCEs remain widely used, and generally apply good assessment practices, such as blueprinting to curricula and the used of valid and reliable instruments [38, 39], but their ability to reflect authentic clinical tasks is less clear.

An observational study was conducted to objectively evaluate students’ communication skills in different learning environments. The study aimed to explore the extent to which the communication skills demonstrated by students in classroom, clinical, and assessment settings align.

## Methods

### Study design

A concurrent triangulation mixed methods study was conducted to observe and evaluate students during the fourth year of a five-year medical program. Concurrent triangulation designs leverage both qualitative and quantitative data collection to enhance the accuracy of defining relationships between variables of interest [40]. Video-recordings were employed during structured classroom ‘interactional skills’ sessions (ISS) or workshops, clinical encounters with patients and an OSCE station requiring communication skills. The use of video recording aimed to ensure the objectivity of data collection and eliminate researcher-participant interaction biases [41]. As communication skills comprise verbal and non-verbal behaviour, video-recording is considered the most suitable method to capture this behaviour alongside their contextual settings [42]. Additionally, field notes were taken during each observation to provide further context to during data analysis. This study was approved by both the University and Health District Human Research Ethics Committees (H-2018-0152 and 2018/PID00638).

### Study sites and participants

This study was undertaken in a five-year undergraduate medical program which includes a structured communication skills curriculum grounded in the principles delineated in the Calgary-Cambridge guide to the medical interview. The curriculum is structured to initiate students into communication micro-skills in the context of classroom-based simulation early in the program, with ongoing opportunities to practise and master these throughout the program during both classroom sessions and application in clinical practice. Interactional skills workshops (classroom) occur throughout the program to align with other curricula elements and clinical rotations. Beginning in the third year, students engage in regular interaction with real patients across various clinical contexts, complemented by continued participation in scheduled interactional skills workshops within the classroom environment. For example, during the Women’s, Adolescent’s, and Children’s Health (WACH) rotation, students attend four structured classroom workshops tailored to the communication skills pertinent to specific clinical scenarios;1) Addressing sensitive issues with adolescents, 2) Partnership with parents, 3) Prenatal screening, and 4) Cervical screening discussions with Aboriginal and Torres Strait Islander women. Evaluation of communication skills is conducted using OSCEs at pivotal points throughout the program.

For the purpose of this study, all fourth-year medical students were invited to participate during their 12-week WACH rotation. This rotation spans clinical placements across five clinical schools in the medical school footprint. The WACH rotation encompasses a blended learning approach, both didactic and clinical components. Students are afforded opportunities to apply communication skills acquired in classroom settings to clinical setting and are assessed in a multi-station OSCE at the end of the rotation. Participation invitations were extended to all students actively enrolled in the course during the designated study period.

### Study procedure

Students received an email invitation from the school administration at the beginning of the rotation, and the study was also briefly described during a lecture prior to clinical placement. Students who consented were observed during an interactive workshop session involving role-play with simulated patients, one real patient encounter, and one end-of-semester OSCE station related to communication skills. As part of this rotation, students attended four ISS workshops focusing on communication skills required in specific situations. They were also expected to keep a record of experience and achievement towards their core clinical competencies, including history-taking and patient communication tasks. Skills were assessed in a multiple station OSCE at the end of semester. Participating students received an AU$20 gift vouchers as appreciation for their time.

### Context of the observation

In-class activities were directly observed and video-recorded, with equipment set up to record role-play interactions between consenting students and a simulated patient. Sessions included eight to twelve students, beginning with discussion of the topic before inviting students to practice skills with simulated patients.

The learning process typically commenced with an introductory overview of the topic, followed by a discussion of students’ clinical rotation experiences. Subsequently, the session advanced to simulated scenarios, wherein various students engaged in role-playing activities. The classroom facilitator initiated each role-play by delineating the scenario and ensuring that students were adequately briefed on their roles and the context before inviting volunteers to interact with the simulated patient. The length of time each student spent in the ‘hot-seat’ engaged in a role-play varied depending on the nature of the session, the facilitator style, and the section of the scenario each student was role-playing.

Clinical educators or health behaviour scientists facilitated the workshops, guided by facilitator instructions which encouraged application of agenda-led, outcome-based analysis of the role-play experiences [19]. As part of the learning process, some students started the role-play at a mid-point of the consultation, picking up from where previous students paused. They continued the conversation whenever previous students left the role. Therefore, not all micro-skills in the CCOG could be observed in every student’s role-play.

The clinical observations were scheduled for times and locations convenient for the participants, either with clinical supervisor during unstructured clinical time, or self-study, usually in Internal Medicine, Paediatrics, or Obstetrics and Gynaecology wards. In this setting, the students aimed to independently take a complete medical history from a patient. Stable and cooperative patients were identified by attending physicians or nurses who sought initial consent for students to approach them. Students also sought permission when approaching patients, to have the consultation recorded for the purpose of the study. The researcher was available to explain the study to the patients if needed. After observing a real patient encounter, a structured debriefing was conducted and recorded for used in the analysis.

One station in which communication was directly assessed (included as one or more items in the marking schema), was observed during an end-of-semester OSCE. Each student had a maximum of eight minutes to respond to a clinical task, after two-minutes reading and preparation time. Students interacted with a simulated patient and examiner based on the task given. In this study, students were observed in three end-of-semester OSCEs with three different cases. All cases related to the women’s health clinical rotation; the first case required the student to discuss a pregnancy test result with a female patient. The second asked the student to discuss contraceptive options with a teenage girl. The third case required the students to discuss urinary incontinence due to uterine prolapse with a female patient. Each simulated patient was trained to present with specific symptoms in response to the students’ questioning. The examiners observed the students and rated their performance based on pre-determined marking criteria. The OSCEs were video recorded without the researcher present.

### Outcome measures/instruments

Students’ communication skills in each context were independently observed and rated using the CCOG [19] by two observers. This evaluation tool has good validity and reliability for evaluating communication skills across a range of settings [17, 19, 43], with moderate intraclass correlation coefficients for each item, ranging from 0.05 to 0.57 [43]. CCOG evaluates six essential communication skills tasks including initiating the session, gathering information, providing structure, building relationships, explanation and planning, and closing the session, and overall performance in interpersonal communication [19]. Each task consists of two to four micro-skills. Not all tasks could be applied to each observation and setting depending on the presenting complaint, the purpose of encounters, and the patient context [19]. Each student’s performance of micro-skills was evaluated using a 3-point Likert scale: “0” (did not perform the skill), “1” (skill was partially performed or not performed well), “2” (skills performed well), and “NA” (not applicable). Overall performance was evaluated using a 9-point Likert scale (1–3 = unsatisfactory, 4–6 = satisfactory, and 7–9 = superior) [44].

During observations, the researcher took field notes which included the context, time and setting of the observation, number and type of attendees (students, facilitator, simulated or real patient), how the sessions occurred, interactions among attendees, and critical reflections of the researcher.

### Analysis method

This study implemented a combination of descriptive quantitative and qualitative approaches. This method uses qualitative data to support and enable a deeper understanding and interpretation of the quantitative data. A concurrent triangulation method was used to analyse data collected from observations. For quantitative data three researchers independently rated a sample of the recordings and reached agreement on ratings before scoring was completed by the lead author. This process ensured that the ratings were representative of the meaning of the task and confirmed that the rating of the data was consistent. The researchers discussed the scores to check for consistency and inter-rater reliability and Cohen’s kappa was calculated [45] as 0.88 (SE = 0.12; CI 95% = 0.65–1.00). SPSS Statistics for Windows (IBM SPSS Statistics for Windows, Version 26.0. Armonk, NY, USA) was used to calculate descriptive statistics for demographic variables and scoring. Analysis of variance was conducted to analyse the mean difference between each setting.

Field notes of observation and video-observation were used to support the description of the findings from quantitative data,

Criteria from CCOG was used to identify themes and provide additional explanatory variances capturing the meaning of data. Iterative process was conducted with frequent discussions among the researchers to ensure agreement and consistency in analysing. A reflection on how these data might influence the research questions and findings, as well as the theoretical interest of the study, accompanied this process.

## Results

Thirty-three students initially agreed to participate; 14 students were observed in all three settings and 14 students had incomplete observations. Five students withdrew from this study – one due to moving clinical schools and being unable to arrange observation and four students withdrew after one observation was conducted. These withdrawals were associated with challenges scheduling further observations and other undisclosed personal reasons. A total of 63 unique observations were included in the final analysis. Table 1 summarises the demographic characteristics of the participants and the number of observations in each setting. Table 2 provides observation time in each setting, and Table 3 shows the average score of students’ performance on each communication skill task.

**Table 1:** Characteristics of participants and observations

	Female	Male	Total
Direct entry from secondary high school	12	12	24
Study/work at health sector	1	3	4
Observation in the classroom only	3	4	7
Observation in the classroom & clinical settings	0	2	2
Observation in the classroom & OSCE settings	2	2	4
Observation in the clinical & OSCE settings	0	1	1
Observation in all settings	8	6	14
**Total**	**13**	**15**	**28**

**Table 2:** Observation time in each setting

	Mean	Standard deviation	Minimal time	Maximal time
Classroom	00:07:13	00:04:04	00:02:25	00:19:42
Clinical	00:18:55	00:08:39	00:07:00	00:35:00
OSCE	00:08:07	00:00:16	00:07:32	00:08:32

**Table 3:** Average scores of student performance based on communication skills tasks of the Calgary-Cambridge Observation Guide

No	Communication skills task	Classroom	Clinical	OSCE	*p*
1	**Initiating the session**	1.4	1.4	1.2	0.43
1.1	Establishing initial report	1.5	1.8	1.3	
1.2	Identifying the reason(s) for the consultation	1.2	1.3	1.1	
2	**Gathering information**	1.2	1.3	1.0	0.50
2.1	Exploration of patient’s problems	1.2	1.4	1.0	
2.2	Additional skills for understanding the patient’s perspective	1.2	0.8	0.7	
3	**Providing structure**	0.5	0.9	1.0	0.005
3.1	Making organisation overt	0.3	0.8	0.6	
3.2	Attending to flow	0.6	1.1	1.3	
4	**Building relationship**	1.2	1.3	1.1	0.26
4.1	Using appropriate non-verbal behaviour	1.4	1.5	1.4	
4.2	Developing rapport	1.4	1.4	1.1	
4.3	Involving the patient	0.6	0.4	0.3	
5	**Explanation and planning**	0.9	NA	0.8	0.33
5.1	Providing the correct amount and type of information	0.9	NA	1.0	
5.2	Aiding accurate recall and understanding	0.7	NA	0.8	
5.3	Achieving a shared understanding: incorporating the patient’s perspective	1.1	NA	0.7	
5.4	Planning: shared decision-making	1.0	NA	0.9	
6	**Closing the session**	1.4	0.7	NA	0.02
6.1	Forward planning	1.5	0.0	NA	
6.2	Ensuring appropriate point of closure	1.8	0.8	NA	
	**Overall communication skills performance**	4.2	4.3	4.2	

(Maximum score for each task: 2.0; Maximum score for overall performance: 9.0)

The overall quantitative performance of students did not differ significantly across settings. The average score for the overall performance in classroom, clinical and OSCE settings was 4.2, 4.3 and 4.2, respectively corresponds to performances which were satisfactory and appropriate for their study level. Nine of 14 students (64%) with complete observations received the same classification (satisfactory) for all settings. Further analysis showed that the performances in the separate components were not statistically significantly different across settings, except for providing structure and closing the session (*p* = 0.005 and *p* = 0.02, respectively). Key differences were found, however, in specific areas of the communication micro-skills across learning environments and this was probably due to the different opportunities to demonstrate skills. (See Supplement material for more detailed micro-skills scores).

We explored the observations from both quantitative and qualitative perspective, considering both scores and rating on the CCOG, and descriptions of the observation themselves. Students started the consultation by *establishing initial rapport*, and *identifying the reason(s) for the consultation.* In the classroom, some students did not perform these tasks as thoroughly as would be expected in a real clinical encounter, in part because many began the role-play as a follow-on from a peer. In the clinical environment, students had longer and unhurried discussions with patients and the tasks associated with initiating the session were performed well. During OSCE, students often rushed to enter the room, sanitise their hands, and greet the patient before they had even reached their chair.

Across all settings, students effectively gathered information by exploring the patient’s problem. Attentive listening and facilitation of patient responses were evident, especially in classroom and clinical environments. They were able to *encourage [the] patient to tell her/his story of the problem, use open and closed questioning techniques*, and *use concise, easily understood questions and comments.* Students also demonstrated an ability to *listen attentively and facilitate patient’s responses appropriately*, particularly in classroom and clinical settings. In the classroom, students used additional skills for understanding the patient’s perspective by *exploring patient’s ideas, concerns, expectation, effects*, *and feelings*, in the clinical setting these micro-skills were observed only in three out of the seventeen clinical observations. In the OSCE, only one of sixteen students performed well in this area (average scores were 1.5, 0.8 and 0.7, respectively in classroom, clinical settings and OSCE). During OSCEs, students tended to rush taking the history of the patient’s problem, primarily using closed-questions in order to complete the OSCE task.

Students generally applied micro-skills of attending to flow to provide the structure of the interview. They *structured interview[s] in a logical sequence* and *attended to timing and kept the interview on task*. However, the micro-skill of using signposting or transitional statements was only observed in seven students (25.9%) in the classroom setting. The nature of classroom role-plays in which students swap roles with their peers likely limited the use of transitional statements, as transitions were often used as a point to pause and move to another student. In contrast, in the clinical setting, students tried to follow patients’ cues to make the interactions flow conversationally, despite using a standard history-taking template. In addition, students were rarely observed *summarising at the end of a specific line of inquiry to confirm understanding before moving on to the next section*. During the OSCE, students rarely structured their consultations with only four of the 16 students *summarising information gathered from the patient*.

The tasks of *building a relationship* were demonstrated consistently in classroom and clinical settings, but less so in the OSCE setting. Students were able to *demonstrate appropriate verbal and non-verbal behaviour (eye contact, facial expression, vocal volume, and tone)* and *develop rapport.* However, micro-skills of *involving the patient* which include *share thinking with a patient to encourage patient’s involvement* were rarely observed in any setting.

The tasks of “Explanation and planning” were observed in the classroom and OSCE settings, but not in the clinical environment. It is not appropriate for students to independently make diagnoses or plan clinical management for real patients. The focus of the interaction in the clinical environment was eliciting a patient’s history to develop clinical reasoning and communication skills. Only a minority of students in the classroom setting had the opportunity to practise the counselling components of a consultation, therefore, their ability to demonstrate *explanation and planning* and to *involve the patient* was rarely observed in their role-play. In the OSCE, this was an expected as part of the assessment.

In classroom observations, most of the students could not *close the session* because the role-plays were stopped by the facilitators before this point. Those who had the opportunity to practise these skills in classroom effectively *contracted with the patient, the next steps for both the patient and physician* and made a *final check that the patient agreed and was comfortable with the plan.* While in the clinical setting, after eliciting a history from the patient, the students closed the session by summarising the information gathered and expressing their gratitude to the patients. In the OSCE, due to the time limitation, none of the students was able to *close the session.* Rather, they rushed to leave the room when the time was over.

Only two of the encounters observed in the clinical environment were also observed by clinical facilitators, and the feedback provided was focused on medical knowledge. Debrief discussions with students suggest that this was reflective of the low level of observation experienced overall. On the other hand, in the classroom settings, the facilitators provided feedback mostly about students’ communication skills, while during the OSCE no feedback was provided to the students.

## Discussion

### Main findings

This study observed 28 students applying communication skills in different learning environments, including assessment. The results highlighted disparities in the practice and focus of skills across settings. The findings revealed that in the classroom students can practise a broad set of communication skills tasks (though not usually each student in a single role-play), however, in the clinical environment, information-gathering and relationship-building with patients were the focus of their encounters. In the OSCEs, limited time and high-pressure scenarios caused the students to solely focus on information-gathering and/or explanation, diminishing opportunity for rapport-building with the patient. These findings indicated a poor alignment between the skills practiced across learning environments. While quantitative differences in communication skills between settings were not statistically significant, important differences emerged in the patterns of skills displayed and components of the consultation practiced in each setting.

The study revealed a disconnection between structured communication skills learned in classrooms and experiences during clinical placements. Simulation-based learning in classrooms offered a safe space for difficult conversations and feedback, aiding preparation for real patient interactions, as also reported elsewhere [46]. Students view simulated patient interaction as a valuable opportunity to prepare themselves for real patient interactions, especially with the ability to “pause” whenever they encounter difficulties [47]. The classroom could fill in an important gap because students cannot appropriately perform many of the more complex tasks with real patients.

The students who participated in this study were trained in *using open and closed questioning techniques, listening attentively, facilitating patient’s responses verbally and non-verbally, picking up verbal and non-verbal cues, using concise, easily understood questions and comments*, and *determining and exploring patient’s ideas, concerns, expectations, and feelings* in the classroom setting. The ability to apply these skills is crucial in patient-centred care and contributes to developing relationships with patients [48]. Yet, these skills were less evident in assessment. Limited time and high-pressure scenarios used in OSCEs restricted students’ opportunity to explore these aspects in the assessment context. It seems that this type of assessment risks devaluing these skills and limits the authenticity of assessment by breaking skills into artificial components rather than assessing them as part of an integrated whole.

Students can practise many communication skills in classroom sessions, but not all tasks can be rehearsed in other learning environments, as also reported in other studies [32, 33, 49]. In the classroom setting, explanation and planning tasks were disseminated across several students performing role-play. On the other hand, students were not required to perform these tasks in the clinical environment. Undergraduate medical students do not have direct responsibility for comprehensive patient care [50, 51]. Their interactions with real patients are conducted under the (often indirect) supervision of attending physicians who act as clinical facilitators and have clinical responsibility. Despite this, the explanation and planning task was evaluated during OSCEs. Although students were able to demonstrate adequate knowledge, they did not use a structured explanation approach based on CCOG while delivering information. This OSCE station, in keeping with those used commonly in medical education programs [22, 52], involved limited time to complete complex clinical tasks. These findings again indicate the misalignment of teaching and assessment, particularly in the communication task of explanation and planning.

Clinical encounters facilitated students’ information-gathering skills and clinical reasoning [46]. However, limited observations by clinical facilitators during these encounters might have hindered their true benefits [32, 49]. In this study, only two clinical encounters were observed by clinical facilitators. In this setting, communication skills are learnt mainly through role-modelling by the supervisors. Patient-centred communication skills learnt in the classroom can be diminished during clinical rotation when students face barriers, such as inconsistent modelling of communication skills by clinicians and other health professions in clinical environments [53–57].

The process of feedback and reflection is helpful to consolidate skills during training [58–61]. Specific feedback on communication skills is suggested to improve students’ ability to handle a patient’s emotions and perceptions, as well as the structure and end of the conversation [59]. A Cochrane review reported that while most educational interventions can have positive impacts on communication skills measured in the short-term post intervention, those involving specific, personalised feedback are likely to have the most impact [8].

In this study, the feedback from facilitators was common, tailored, and received well during classroom sessions. However, not all students had the opportunity to practise with a simulated patient and receive personalised feedback on their performance. Only half of the students in one workshop interacted with simulated patients while the remainder only observed the interactions. Feedback was very limited in the clinical environment, with only two clinical encounters observed by clinical facilitators, and the feedback focused on medical knowledge. In summative OSCEs, feedback was limited to the assessment outcome or grade. While the examiners rated student’s performance based on pre-determined marking criteria, the majority of points were related to the medical knowledge and management of the case, with only one of ten aspects evaluating students’ communication skills. It is therefore possible for a student to pass an OSCE without establishing any rapport with a patient or involving the patient in the consultation. Such a student is receiving a message which is likely to be very different from the feedback they would receive on the same performance in a classroom session. Again, it shows the discrepancy of teaching and learning in different settings.

The style and type of feedback provided shows substantial discrepancy across learning environments, in keeping with the previous literature [49]. Inconsistent or absent feedback received during clinical rotations can be counterproductive and reinforce poor practices [20]. This practice leads to a misalignment between students’ understanding of good communication skills, based on the models they observe and practise in clinical environments, and what they are expected to do in an OSCE that focuses more on content than the interview process, as discussed in other study [36].

Clinical communication skills learning not only teaches students a medical consultation structure but also how and when to apply it in different contexts, using the micro-skills that increase efficacy [62]. Students need to gradually learn from simulated patient interactions and real patient encounters, in increasingly complex cases [63, 64] to be able to develop the flexibility and capability to apply their communication skills appropriately to patients in different contexts [65]. However, the misalignment of learning and assessment illustrated in this study may contribute to the difficulty of applying communication skills across learning environments.

### Limitations of the study

This study involved a group of students in the same year of a single undergraduate entry medical program, potentially limiting the generalisability of findings to other programs or different student cohorts. However, the nature of clinical and assessment experiences is reasonably consistent across medical programs [66, 67]. The voluntary nature of participation might skew results towards a more motivated and confident group, not necessarily representative of the whole cohort. However, the mix of students with regard to gender, age and background lends weight to the validity of these observational data.

This study observed students on a single occasion for every setting. It did not follow the students to the following year to confirm whether the skills remained or changed over a more extended period. In OSCEs, the case used might not be the most suitable case to evaluate these skills. Each of the students observed in OSCEs were marked as having at least met the required standard for the station as a whole. In addition, using video observation might cause observer effect, observer bias and observer expectation. Since the students were aware of being observed, they might have performed better than normal or shown a “halo effect” [41]. However, video observations in this study captured verbal and non-verbal behaviour during the encounter and could be replayed for rating purposes. Evaluation of students using longitudinal observation across settings during their regular learning environments might better capture student performance.

## Conclusion

This study observed students during communication skills learning and assessment. Not all aspects of communication skills can be practised in all learning environments. Classroom workshops attempt to cover every aspect of communication skill, often spread across several students. In contrast, in the clinical environment, students focus mainly on information-gathering, while in the OSCE, students are often tasked with performing an isolated task such as gathering a history, or explanation and planning with a simulated patient. Students are required to build a relationship in all settings, but in the eight-minute OSCE this is particularly challenging. The differences between these learning and assessment settings mean that students do not receive clear messages about what is valued and prioritised in terms of clinical communication.

The critical components of role-play practice, feedback, observation, and supervision are well-acknowledged, but the quality of application of each of these components differs across learning environments. The misalignment of teaching and assessment may contribute to students’ confusion when transferring their communication skills to different learning environments. Students would benefit from opportunities to rehearse and practise in different learning environments and receive feedback on their performance in each setting to help them transfer the skills across learning environments and develop their flexibility and capability. Combining formal communication skills in classroom sessions with experiential learning during clinical rotations, coupled with observation and feedback, may be an effective approach to develop an understanding of both the theoretical content and practical application of communication skills. However, the efficacy of this approach hinges on the alignment of teaching, learning and assessment of communication skills across learning environments, including the role-modelling of communication skills by clinicians. Further research is needed to investigate the development and application of students’ skills over the long term to understand supports for and barriers to effective teaching and learning of doctor-patient communication skills in different learning environments.

### Electronic supplementary material

Below is the link to the electronic supplementary material.

Supplementary Material 1

## Data Availability

Our data are available in the Open Science Framework repository at https://osf.io/5xayz/.https://osf.io/ezqh6/files/osfstorage/655d581e062a3e3da1ee05fb.

## Abbreviations

CCOG: Calgary-cambridge observation guide
ISS: Interactional skills’ sessions
JMP: Joint medical program
OSCE: Objective structured clinical examination
WACH: women’s, adolescents’ and children’s health
