# Supplementary material for: Do the teaching, practice and assessment of clinical communication skills align?
Source: BMC Med Educ. 2024 Jun 1;24:609. doi: 10.1186/s12909-024-05596-8 (PMC11144343; doi:10.1186/s12909-024-05596-8)
Supplement: Supplementary file 1 — Supplementary Material 1 [file 12909_2024_5596_MOESM1_ESM.docx]

**Supplement material: The average score of micro-skills based on Calgary-Cambridge Observation Guide**

| **No.** | **Micro-skill to be observed** | **Classroom** | | | | **Clinical** | | | | **OSCE** | | |
| --- | --- | --- | --- | --- | --- | --- | --- | --- | --- | --- | --- | --- |
|  |  | **Mean** | **Median** | **Modus** | **Mean** | | **Median** | **Modus** | **Mean** | | **Median** | **Modus** |
| **1** | **Initiating the session** | **1.4** | **1.3** | **1.3** | **1.4** | | **1.6** | **1.7** | **1.2** | | **1.2** | **1.3** |
| 1.1 | Establishing initial report | 1.5 | 1.5 | 1.5 | 1.8 | | 1.8 | 2.0 | 1.3 | | 1.2 | 1.0 |
| 1 | Greets patient and obtains patient’s name | 1.7 | 2.0 | 2.0 | 1.9 | | 2.0 | 2.0 | 1.3 | | 1.0 | 2.0 |
| 2 | Introduces self, role, and nature of interview; obtain consent if necessary | 1.2 | 1.0 | 1.0 | 1.9 | | 2.0 | 2.0 | 1.4 | | 1.0 | 1.0 |
| 3 | Demonstrates respect and interest, attends to patient’s physical comfort | 1.3 | 1.0 | 1.0 | 1.5 | | 1.5 | 2.0 | 1.2 | | 1.0 | 1.0 |
| 1.2 | Identifying the reason(s) for the consultation | 1.2 | 1.3 | 1.3 | 1.3 | | 1.3 | 1.3 | 1.1 | | 1.0 | 0.8 |
| 4 | Identifies the patient’s problem or the issues that the patient wishes to address with appropriate opening question (e.g., “What problems brought you to the hospital?” or “What would you like to discuss today?” or “What questions did you hope to get answered today?” | 1.7 | 2.0 | 2.0 | 1.8 | | 2.0 | 2.0 | 1.6 | | 2.0 | 2.0 |
| 5 | Listens attentively to the patient’s opening statement, without interrupting or directing patient’s response | 1.5 | 2.0 | 2.0 | 1.6 | | 2.0 | 2.0 | 1.3 | | 1.0 | 2.0 |
| 6 | Confirm list and screens for further problems (e.g., “so that’s headaches and tiredness; anything else….?) | 0.6 | 0.0 | 0.0 | 0.9 | | 1.0 | 0.0 | 0.6 | | 0.0 | 0.0 |
| 7 | Negotiate agenda taking both patient’s and physician’s need into account | 1.0 | 1.0 | 0.0 | 0.1 | | 0.0 | 0.0 | 1.0 | | 1.0 | 2.0 |
| **2** | **Gathering information** | **1.2** | **1.2** | **1.5** | **1.3** | | **1.4** | **1.5** | **1.0** | | **1.0** | **0.6** |
| 2.1 | Exploration of patient’s problems | 1.2 | 1.2 | 1.8 | 1.4 | | 1.4 | 1.9 | 1.0 | | 1.1 | 0.7 |
| 8 | Encourages patient to tell the story of the problem(s) from when first started to the present in own words (clarifying reason for presenting now) | 1.5 | 2.0 | 2.0 | 1.8 | | 2.0 | 2.0 | 1.1 | | 1.0 | 1.0 |
| 9 | Uses open and closed questioning technique, appropriately moving from open to closed | 1.5 | 2.0 | 2.0 | 1.6 | | 2.0 | 2.0 | 1.0 | | 1.0 | 1.0 |
| 10 | Listens attentively, allowing patient to complete statements without interruption and leaving space for patient to think before answering or go on after pausing | 1.6 | 2.0 | 2.0 | 1.6 | | 2.0 | 2.0 | 1.4 | | 1.0 | 1.0 |
| 11 | Facilities patient’s responses verbally and non-verbally e.g., use of encouragement, silence, repetition, paraphrasing, interpretation | 1.3 | 1.0 | 1.0 | 1.5 | | 2.0 | 2.0 | 1.1 | | 1.0 | 1.0 |
| 12 | Picks up verbal and non-verbal cues (body language, speech, facial expression, affect); checks out and acknowledge as appropriate | 1.1 | 1.0 | 1.0 | 1.4 | | 1.0 | 1.0 | 1.1 | | 1.0 | 1.0 |
| 13 | Clarifies patient’s statements that are unclear or need amplification (e.g., “Could you explain what you mean by lightheaded”) | 0.8 | 1.0 | 0.0 | 1.0 | | 1.0 | 2.0 | 0.6 | | 0.5 | 0.0 |
| 14 | Periodically summaries to verify own understanding of what the patient has said, invites patient to correct interpretation or provide further information | 0.3 | 0.0 | 0.0 | 0.6 | | 0.0 | 0.0 | 0.2 | | 0.0 | 0.0 |
| 15 | Uses concise, easily understood questions and comments, avoids, or adequately explains jargon | 1.7 | 2.0 | 2.0 | 1.9 | | 2.0 | 2.0 | 1.7 | | 2.0 | 2.0 |
| 16 | Establishes dates and sequence of events | 0.7 | 0.5 | 0.0 | 1.6 | | 2.0 | 2.0 | 0.8 | | 1.0 | 1.0 |
| 2.2 | Additional skills for understanding the patient’s perspective | 1.2 | 1.5 | 2.0 | 0.8 | | 1.0 | 1.0 | 0.7 | | 0.8 | 1.0 |
| 17 | Actively determines and appropriately explores:  - Patient’s ideas (i.e., beliefs or cause)  - Patient’s concerns (i.e., worries) regarding each problem  - Patient’s expectation (i.e., goals, what help the patient had expected for each problem)  - Effects: how each problem affects the patient’s life | 1.3 | 1.0 | 2.0 | 0.9 | | 1.0 | 1.0 | 0.9 | | 1.0 | 1.0 |
| 18 | Encourages patient to express feelings | 1.2 | 1.5 | 2.0 | 0.7 | | 1.0 | 1.0 | 0.6 | | 0.5 | 0.0 |
| **3** | **Providing Structure** | **0.5** | **0.5** | **0.0** | **0.9** | | **0.8** | **0.8** | **1.0** | | **0.9** | **0.8** |
| 3.1 | Making organisation overt | 0.3 | 0.0 | 0.0 | 0.8 | | 0.5 | 0.5 | 0.6 | | 0.5 | 1.0 |
| 19 | Summarises at the end of specific line of inquiry to confirm understanding before moving on to the next section | 0.3 | 0.0 | 0.0 | 0.5 | | 0.0 | 0.0 | 0.3 | | 0.0 | 0.0 |
| 20 | Progresses from one section to another using signposting, transitional statements; includes rationale for next section | 0.3 | 0.0 | 0.0 | 1.1 | | 1.0 | 1.0 | 0.9 | | 1.0 | 1.0 |
| 3.2 | Attending to flow | 0.6 | 0.5 | 0.0 | 1.1 | | 1.0 | 1.0 | 1.3 | | 1.3 | 1.0 |
| 21 | Structures interview in logical sequence | 0.7 | 1.0 | 0.0 | 1.1 | | 1.0 | 1.0 | 1.4 | | 1.5 | 2.0 |
| 22 | Attends to timing and keeping interview on task | 0.7 | 1.0 | 1.0 | 1.1 | | 1.0 | 1.0 | 1.2 | | 1.0 | 1.0 |
| **4** | **Building Relationship** | **1.2** | **1.1** | **2.0** | **1.3** | | **1.4** | **1.0** | **1.1** | | **1.0** | **1.0** |
| 4.1 | Using appropriate non-verbal behaviour | 1.4 | 1.5 | 2.0 | 1.5 | | 1.5 | 2.0 | 1.4 | | 1.3 | 1.3 |
| 23 | Demonstrate appropriate non-verbal behaviour  - Eye contact, facial expression  - Posture, position, and movement  - Vocal cues e.g., rate, volume, tone | 1.6 | 2.0 | 2.0 | 1.6 | | 2.0 | 2.0 | 1.8 | | 2.0 | 2.0 |
| 24 | If reads, writes notes or uses computer, does in a manner that does not interfere with dialogue or rapport | 0.8 | 1.0 | 1.0 | 1.3 | | 1.0 | 2.0 | 1.3 | | 1.0 | 2.0 |
| 25 | Demonstrates appropriate confidence | 1.3 | 1.0 | 1.0 | 1.4 | | 1.0 | 2.0 | 1.3 | | 1.0 | 1.0 |
| 4.2 | Developing rapport | 1.4 | 1.5 | 2.0 | 1.4 | | 1.5 | 1.8 | 1.1 | | 1.0 | 1.0 |
| 26 | Accepts legitimacy of patient’s views and feelings; is not judgmental | 1.4 | 2.0 | 2.0 | 1.4 | | 2.0 | 2.0 | 1.1 | | 1.0 | 1.0 |
| 27 | Uses empathy to communicate understanding and appreciation of the patient’s feelings or predicament; overtly acknowledge patient’s views and feelings | 1.4 | 2.0 | 2.0 | 1.5 | | 1.0 | 1.0 | 1.0 | | 1.0 | 1.0 |
| 28 | Provides support: express concern, understanding, willingness to help; acknowledges coping efforts and appropriate self-care; offers partnership | 1.4 | 2.0 | 2.0 | 1.6 | | 2.0 | 2.0 | 1.1 | | 1.0 | 1.0 |
| 29 | Deals sensitivity with embarrassing and disturbing topics and physical pain, including when associated with physical examination | 1.1 | 1.0 | 1.0 | 1.1 | | 1.0 | 1.0 | 1.3 | | 1.0 | 1.0 |
| 4.3 | Involving the patient | 0.6 | 0.0 | 0.0 | 0.4 | | 0.3 | 0.0 | 0.3 | | 0.0 | 0.0 |
| 30 | Shares thinking with patient to encourage patient’s involvement (e.g., “What I’m thinking now is….”) | 0.5 | 0.0 | 0.0 | 0.1 | | 0.0 | 0.0 | 0.2 | | 0.0 | 0.0 |
| 31 | Explain rationale for questions or parts of physical examination that could be non-sequiturs | 0.6 | 0.0 | 0.0 | 0.6 | | 0.5 | 0.0 | 0.5 | | 0.0 | 0.0 |
| 32 | During physical examination, explain process, asks permission | 0.0 | 0.0 | #N/A | 1.5 | | 1.5 | #N/A | #N/A | | #N/A | #N/A |
| **5** | **Explanation and planning** | **0.9** | **1.1** | **1.3** | **#N/A** | | **#N/A** | **#N/A** | **0.8** | | **0.7** | **0.3** |
| 5.1 | Providing the correct amount and type of information | 0.9 | 1.0 | 1.0 | #N/A | | #N/A | #N/A | 1.0 | | 0.9 | 0.8 |
| 33 | Chunks and checks: give information in manageable chunks, checks for understanding, uses patient’s response as guide to how to proceed | 0.2 | 0.0 | 0.0 | #N/A | | #N/A | #N/A | 0.7 | | 1.0 | 0.0 |
| 34 | Assesses patient’s starting point asks for patient’s prior knowledge early on when giving information, discovers extent of patient’s wish for information | 1.2 | 1.0 | 1.0 | #N/A | | #N/A | #N/A | 0.9 | | 1.0 | 1.0 |
| 35 | Asks patients what other information would be helpful e.g., aetiology, prognosis | 1.0 | 1.0 | 1.0 | #N/A | | #N/A | #N/A | 1.1 | | 1.0 | 2.0 |
| 36 | Gives explanation at appropriate times avoids giving advice, information, or reassurance prematurely | 1.3 | 1.5 | 2.0 | #N/A | | #N/A | #N/A | 1.1 | | 1.0 | 1.0 |
| 5.2 | Aiding accurate recall and understanding | 0.7 | 0.8 | 0.3 | #N/A | | #N/A | #N/A | 0.8 | | 0.8 | 0.7 |
| 37 | Organises explanation: divides into discrete sections, develops a logical sequence | 0.3 | 0.0 | 0.0 | #N/A | | #N/A | #N/A | 1.0 | | 1.0 | 1.0 |
| 38 | Uses explicit categorisation or signposting (e.g., “There are three important things that I would like to discuss. 1st….” “Now, shall we move on to…”) | 0.4 | 0.0 | 0.0 | #N/A | | #N/A | #N/A | 0.6 | | 0.5 | 0.0 |
| 39 | Uses repetition and summarising to reinforce information | 0.3 | 0.0 | 0.0 | #N/A | | #N/A | #N/A | 0.4 | | 0.0 | 0.0 |
| 40 | Uses concise, easily understood language, avoids, or explains jargon | 1.5 | 2.0 | 2.0 | #N/A | | #N/A | #N/A | 1.7 | | 2.0 | 2.0 |
| 41 | Uses visual methods of conveying information: diagrams, models, written information, and instruction | 0.2 | 0.0 | 0.0 | #N/A | | #N/A | #N/A | 0.5 | | 0.0 | 0.0 |
| 42 | Checks patient’s understanding of information given (or plans made): e.g., by asking patient to restate in own words; clarifies as necessary | 1.1 | 1.0 | 1.0 | #N/A | | #N/A | #N/A | 0.4 | | 0.0 | 0.0 |
| 5.3 | Achieving a shared understanding: incorporating the patient’s perspective | 1.1 | 1.0 | 2.0 | #N/A | | #N/A | #N/A | 0.7 | | 0.8 | 0.0 |
| 43 | Relates explanations to patient’s illness framework: to previously elicited ideas, concerns and expectations | 1.0 | 1.0 | 0.0 | #N/A | | #N/A | #N/A | 0.6 | | 0.0 | 0.0 |
| 44 | Provides opportunities and encourages patient to contribute: to ask questions, seek clarification or express doubts; responds appropriately | 1.1 | 1.0 | 1.0 | #N/A | | #N/A | #N/A | 1.0 | | 1.0 | 1.0 |
| 45 | Picks up verbal and non-verbal cues e.g., patient’s need to contribute information or ask questions, information overload, distress | 1.0 | 1.0 | 1.0 | #N/A | | #N/A | #N/A | 0.8 | | 1.0 | 0.0 |
| 46 | Elicits patient’s beliefs, reactions and feelings re information given, terms used; acknowledges and address where necessary | 0.8 | 1.0 | 0.0 | #N/A | | #N/A | #N/A | 0.4 | | 0.0 | 0.0 |
| 5.4 | Planning: shared decision making | 1.2 | 1.3 | 2.0 | #N/A | | #N/A | #N/A | 0.9 | | 0.7 | 0.7 |
| 47 | Shares own thinking as appropriate: ides, thought processes, dilemmas | 1.2 | 1.0 | 1.0 | #N/A | | #N/A | #N/A | 0.6 | | 1.0 | 1.0 |
| 48 | Involves patient by making suggestions rather than directives | 1.5 | 2.0 | 2.0 | #N/A | | #N/A | #N/A | 1.2 | | 1.0 | 2.0 |
| 49 | Encourages patient to contribute their thoughts: ideas, suggestions, and preferences | 1.3 | 2.0 | 2.0 | #N/A | | #N/A | #N/A | 0.9 | | 1.0 | 1.0 |
| 50 | Negotiates a mutually acceptable plan | 0.8 | 0.5 | 0.0 | #N/A | | #N/A | #N/A | 0.6 | | 0.5 | 0.0 |
| 51 | Offers choices encourages patient to make choices and decisions to the level that they wish | 1.2 | 1.0 | 1.0 | #N/A | | #N/A | #N/A | 1.0 | | 1.0 | 1.0 |
| 52 | Checks with patient if accepts plan if concerns have been addresses | 1.2 | 1.5 | 2.0 | #N/A | | #N/A | #N/A | 1.1 | | 1.0 | 2.0 |
| **6** | **Closing the session** | **1.4** | **1.5** | **1.5** | **0.7** | | **1.0** | **1.0** | **#N/A** | | **#N/A** | **#N/A** |
| 6.1 | Forward planning | 1.5 | 1.5 | #N/A | 0.0 | | 0.0 | 0.0 | #N/A | | #N/A | #N/A |
| 53 | Contracts with patient re next steps for patient and physician | 2.0 | 2.0 | 2.0 | 0.3 | | 0.0 | 0.0 | #N/A | | #N/A | #N/A |
| 54 | Safety nets, explaining possible unexpected outcomes, what to do if plan is not working, when and how to seek help | 0.5 | 0.5 | #N/A | 0.0 | | 0.0 | 0.0 | #N/A | | #N/A | #N/A |
| 6.2 | Ensuring appropriate point of closure | 1.8 | 1.8 | #N/A | 0.8 | |  | 1.0 | #N/A | | #N/A |  |
| 55 | Summaries session briefly and clarifies plan of care | 1.3 | 1.0 | 1.0 | 0.7 | | 1.0 | 1.0 | #N/A | | #N/A | #N/A |
| 56 | Final check that patient agrees and is comfortable with plan and asks if any corrections, questions, or other items to discuss | 1.7 | 2.0 | 2.0 | 0.8 | | 1.0 | 1.0 | #N/A | | #N/A | #N/A |
| **7** | **Overall communication skills performance** | 4.2 | 4.0 | 4.0 | 4.3 | | 4.0 | 5.0 | 4.2 | | 4.0 | 4.0 |
|  |  |  |  |  |  | |  |  |  | |  |  |
|  | **OPTIONS IN EXPLANATION AND PLANNING (includes content)** |  |  |  |  | |  |  |  | |  |  |
|  | **IF discussion investigation and procedures** | 1.0 | 1.0 | 1.0 | #N/A | | #N/A | #N/A | #N/A | | #N/A | #N/A |
| 57 | Provides clear information on procedures, e.g., what patient might experience, how patient will be informed of result | 0.9 | 1.0 | 1.0 | #N/A | | #N/A | #N/A | #N/A | | #N/A | #N/A |
| 58 | Relates procedures to treatment plan: values, purpose | 1.1 | 1.0 | 1.0 | #N/A | | #N/A | #N/A | #N/A | | #N/A | #N/A |
| 59 | Encourages questions about and discussion of potential anxieties or negative outcomes | 1.1 | 1.0 | 1.0 | #N/A | | #N/A | #N/A | #N/A | | #N/A | #N/A |
|  | **IF discussing opinion and significance of problem** | 1.3 | 1.3 | #N/A | #N/A | | #N/A | #N/A | 1.0 | | 1.0 | 1.0 |
| 60 | Offers opinion of what is going on and names if possible | 1.3 | 1.0 | 1.0 | #N/A | | #N/A | #N/A | 1.0 | | 1.0 | 1.0 |
| 61 | Reveals rationale for opinion | 1.3 | 1.0 | 1.0 | #N/A | | #N/A | #N/A | 1.0 | | 1.0 | 1.0 |
| 62 | Explains causation, seriousness, expected outcome, short and long-term consequences | 1.0 | 1.0 | #N/A | #N/A | | #N/A | #N/A | 0.5 | | 0.5 | #N/A |
| 63 | Elicits patient’s beliefs, reactions, concerns, re opinion | 1.7 | 2.0 | 2.0 | #N/A | | #N/A | #N/A | #N/A | | #N/A | #N/A |
|  | **IF discussing mutual plan of action** | 1.4 | 1.4 | #N/A | #N/A | | #N/A | #N/A | 1.8 | | 2.0 | 2.0 |
| 64 | Discusses options e.g. No action, investigations, medication or surgery, non-drug treatments (physiotherapy, walking aids, fluids, counselling, preventive measures) | 1.5 | 1.5 | #N/A | #N/A | | #N/A | #N/A | 1.3 | | 1.5 | 2.0 |
| 65 | Provides information on action or treatment offered   - Name - Steps involved, how it works - Benefits and advantages - Possible side effects | 1.0 | 1.0 | #N/A | #N/A | | #N/A | #N/A | #N/A | | #N/A | #N/A |
| 66 | Obtains patient’s view of need for action, perceived benefits, barriers, motivation | 1.5 | 1.5 | #N/A | #N/A | | #N/A | #N/A | 0.8 | | 1.0 | 1.0 |
| 67 | Accepts patient’s view, advocates alternative viewpoint as necessary | 1.5 | 1.5 | #N/A | #N/A | | #N/A | #N/A | 0.5 | | 0.0 | 0.0 |
| 68 | Elicits patient’s reactions and concerns about plans and treatments including acceptability | 1.5 | 1.5 | #N/A | #N/A | | #N/A | #N/A | 0.8 | | 1.0 | 0.0 |
| 69 | Takes patient’s lifestyle, beliefs, cultural background, and abilities into consideration | 1.5 | 1.5 | #N/A | #N/A | | #N/A | #N/A | 0.5 | | 0.0 | 0.0 |
| 70 | Encourages patient to be involved in implementing plans, to take responsibility and be self-reliant | 1.5 | 1.5 | #N/A | #N/A | | #N/A | #N/A | 0.7 | | 1.0 | 0.0 |
| 71 | Asks about patient support systems, discusses other support available | 0.5 | 0.5 | #N/A | #N/A | | #N/A | #N/A | 0.4 | | 0.0 | 0.0 |

(Maximum score for each task: 2.0)
